# Echocardiographic evaluation of cardiovascular complications after birth asphyxia in term neonates

**DOI:** 10.12669/pjms.335.12849

**Published:** 2017

**Authors:** Mohsen Shahidi, Ghodratollah Evazi, Abdorrahim Afkhamzadeh

**Affiliations:** 1 Mohsen Shahidi, Assistant Professor of Pediatric Cardiology, Dept. of Pediatrics, Faculty of Medicine, Kurdistan University of Medical Sciences, Sanandaj, Iran; 2 Ghodratollah Evazi, Pediatric Specialist, Dept. of Pediatrics, Faculty of Medicine, Kurdistan University of Medical Sciences, Sanandaj, Iran; 3 Abdorrahim Afkhamzadeh, Associate Professor of Community Medicine, Dept. of Community Medicine, Kurdistan University of Medical Sciences, Sanandaj, Iran

**Keywords:** Birth asphyxia, Cardiovascular complications, Echocardiographic evaluation, Term neonates

## Abstract

**Objectives:**

After birth asphyxia, a variety of hemodynamic disorders may be noted in the neonatal intensive care unit; these require appropriate recognition and management. The present study was designed to demonstrate the prevalence of heart complications amongst asphyxiated newborns.

**Methods:**

Through a cohort study, 29 asphyxiated term neonates were followed since birth until amelioration of pulmonary hypertension and compared with 31 well born neonates. Both groups were evaluated for their heart anatomy and hemodynamic with meticulous assessment through echocardiography. This study was conducted in Besat Medical Center since August 2010 until February 2012.

**Results:**

Hemodynamic and anatomic disorders including myocardial dysfunction, pulmonary hypertension and patent arterial duct (PDA) were strongly associated with birth asphyxia (P< 0.05).

**Conclusion:**

Birth asphyxia was associated with systolic and diastolic dysfunction and pulmonary hypertension which demands precise evaluation, early recognition and appropriate management.

## INTRODUCTION

Birth asphyxia is defined as metabolic acidemia due to decreased oxygen transfer through the placenta or neonatal respiratory tract with intrapartum pH of less than 7.00 and base deficit greater than 12 mmol/l.^1^ Birth asphyxia results in multi organ failure including neonatal encephalopathy.^1^ Hypoxic ischemic encephalopathy, particularly if associated with fetal acidosis of PH<7 and fifth minute APGAR (Appearance, Pulse, Grimace, Activity, Respiratory) score of 0 to 3, causes more severe organ disorders.^1^,^2^ During severe hypoxia cardiovascular disturbances include tricuspid valve regurgitation which is relatively frequent, mitral valve regurgitation that is less common, pulmonary hypertension and transient myocardial ischemia.^2^-^4^ However, cardiovascular disturbances could be easily ignored because more attention is paid to other end organ disorders. Many reports attempt to demonstrate the importance of early recognition of cardiac dysfunction by using echocardiography or other methods.^3^,^5^,^6^

The aim of this study was to evaluate asphyxiated neonates for possible occurrence of cardiovascular disorders.

## METHODS

Overall, 29 asphyxiated term neonates were followed since birth and compared with 31 well newborns for cardiovascular complications in the neonatal intensive care unit of Besat hospital since August 2010 until February 2012. In both groups first echocardiography was performed in the first or second day after birth. Follow up echocardiography was done at least once a week until improvement of pulmonary hypertension or minimum one month. In a few patients (2 patients) echocardiography evaluation was scheduled for more than one month due to prolongation of pulmonary hypertension. All neonates were between 38 to 41 weeks of gestational age determined by the last menstrual period (LMP) or ultrasound method. Besat hospital is the referral center for the neonatal intensive care in Sanandaj, the capital of Kurdistan province. Asphyxiated neonates were categorized into two groups of moderate and severe types.^7^,^8^

We excluded neonates with congenital heart disease other than patent arterial duct and patent foramen oval from this study. Likewise, patients with congenital or acquired lung disease were omitted. Moreover, patients with other organ anomaly or chromosomal disorders and those with early septicemia were not considered in this study.

Univariate analysis was used to compare variables for the outcome groups of interest. Continuous variables were compared using Student’s *t-* test for normally distributed variables. The Chisquare and Fisher exact test were used to compare categorical variables. All P-values lower than 0.05 were considered significant with SPSS 20. The study was approved by the Ethical Committee of Kurdistan University of Medical Sciences.

## RESULTS

In birth asphyxia group eleven patients (37.9%) were female and eighteen (62%) males. Comparing with healthy controls, although asphyxia was numerically more common in males, it was not statistically significant (P=0.132).

Patent arterial duct was a common finding in asphyxiated neonates (62%), whereas, only one normal newborn had patent arterial duct which shows significant relationship between asphyxia and this complication (P<0.001) (Table I and II). Furthermore, patent arterial duct was strongly correlated with pulmonary hypertension and diastolic dysfunction (P<0.001).

**Table-I:** Comparing asphyxiated and healthy neonates for cardiovascular variables.

*Characteristic*	*Category*	*Asphyxia N (%)*	*Control N (%)*	**P* -Value*	*OR (95% CI)*
Sex	Male	18 (62.1)	13 (41.9)	0.10	-
	Female	11 (37.9)	18 (58.1)		
Patent duct arterial	Yes	18 (94.7)	1 (5.3)	<0.001	0.02 (0.002-0.01)
	No	11 (26.8)	30 (73.2)		
Tricuspid Regurgitation	Yes	29 (64.4)	16 (35.6)	0.003	0.17 (0.05–0.59)
	No	0 (0)	15 (100)		
Mitral Regurgitation	Yes	3 (10)	0 (0)	0.06	-
	No	26 (90)	31 (54.4)		
Pulmonary Hypertension	Yes	20 (76.9)	6 (23.1)	<0.001	0.1 (0.03–0.35)
	No	9 (26.5)	25 (73.5)		
Systolic Dysfunction	Yes	9 (100)	0 (0)	<0.001	0.39 (0.27–0.55)
	No	20 (39.2)	31 (60.8)		
Diastolic Dysfunction	Yes	15 (100)	0 (0)	<0.001	0.31 (0.20–0.48)
	No	14 (31.1)	31 (68.9)		

*Note:* OR: Odds Ratio, CI: confidence interval.

**Table-II:** Comparison of heart complication in three groups of newborns (moderate and severe Asphyxia and healthy children), Sanandaj Be, asat Hospital (Univariate analysis).

*Characteristic*	*Category*	*Asphexia N (%)*	*Control N (%)*	*P Value*	*OR (95% CI)*
Patent duct arterial	No	11 (26.8)	30 (73.2)		-
	Small	6 (100.0)	0 (0.0)	<0.001	1.54 (1.09 – 2.20)
	Moderate to Large	12(92.3)	1 (7.7)	<0.001	32.43 (3.80 – 282.03)
Tricuspid Regurgitation	No	0 (0)	15 (100)		-
	Mild	26	14	0.36	1.07 (0.94 – 1.23)
	Moderate to Severe	3	2	0.005	5.20 (1.55 – 17.44)
Systolic Dysfunction	No	20 (39.2)	31 (60.8)		-
	Moderate	6	0	0.005	1.3 (1.05 – 1.60)
	Severe	3	0	0.039	1.15 (0.98 – 1.35)
Diastolic Dysfunction	No	14 (31.1)	31 (68.9)		-
	Moderate	8	0	0.001	1.50 (1.11 – 2.03)
	Severe	7	0	<0.001	1.57 (1.15 – 2.15)

*Note:* OR: Odds Ratio, CI: confidence interval.

There was a prominent relationship between tricuspid valve regurgitation and asphyxia (P=0.003) (Table-I). On the other hand, the intensity of tricuspid regurgitation was positively correlated with the level of pressure gradient (P=0.003). Consequently, neonatal asphyxia was positively related to increased pulmonary pressure. In addition, there was significant statistical relationship between pulmonary hypertension and diastolic dysfunction (P=0.002). Hence, there would be an association between pulmonary hypertension and cardiac hemodynamic.

Mitral valve regurgitation was relatively rare among the asphyxiated group; however, moderate regurgitation was found in one patient with moderate asphyxia and in two others with severe type (P=0.06) (Table-I).

About one third of asphyxiated neonates had different levels of systolic dysfunction. Statistical assessment showed positive relationship between severity of asphyxia and systolic dysfunction (P=0.01 and 0.001) (Tables I and II). Diastolic dysfunction was detected in about half of the asphyxiated neonates (P<0.001) (Table-I) with relatively equal frequency of “mild to moderate” and “severe” dysfunctions (Table-II). Likewise, diastolic dysfunction was well associated with tricuspid regurgitation, pulmonary hypertension and patent arterial duct (P values of <0.001, <0.001 and <0.001 respectively).

Pulmonary hypertension was present in near two-thirds of asphyxiated neonates. All of our patients with severe asphyxia had different levels of pulmonary hypertension, whereas, pulmonary hypertension was present in 57% of those with moderate asphyxia. Statistical analysis showed significant relationship between increased pulmonary hypertension and asphyxia (P<0.001) (Table-I).

There was no statistical relationship between weight and other variables including patent arterial duct, tricuspid regurgitation, and pulmonary hypertension, systolic and diastolic dysfunction. Antero posterior chest X ray was commonly abnormal in asphyxiated neonates particularly those with severe type (P<0.001) showing increased cardio thoracic ratio or pulmonary vascular marking.

## DISCUSSION

In this study, we tried to evaluate cardiovascular complications after birth asphyxia especially by accurate echocardiographic assessment. Ventricular diastolic dysfunction was present in more than half of the neonates with either moderate or severe asphyxia; however, patients with severe asphyxia had higher grade of diastolic dysfunction. Likewise, systolic dysfunction was statistically affected by asphyxia. The degree of asphyxia determines the severity of cardiac dysfunction as it may be ignorable in mild hypoxia.^4^ Transitory myocardial ischemia is often seen as a complication of severe asphyxia which may range from tachypnea to cardiogenic shock.^9^ Severe asphyxia may cause myocardial dysfunction and injury or ischemic myocardial necrosis in both ventricles as a result of under perfusion.^10^,^11^ Persistent low cardiac output during the first 48 hours of life in newborns with perinatal asphyxia is associated with a significantly higher mortality.^12^ However, asphyxia itself could potentially have adverse outcomes on the neonatal heart; nevertheless, oxidative effect of reoxigenation may cause severe cardiovascular consequences with high morbidity and mortality which is due to degradation of cardiac myosin light chain 1 protein (MLC1) by matrix metalloproteinase-2 (MMP-2).^13^,^14^ However, myocardial ischemia or hypoxia after birth asphyxia is a well-known cause of cardiac dysfunction; nevertheless, other causes such as pulmonary hypertension and patent arterial duct would play an important role.^15^-^17^ We found significant statistical relationship between diastolic dysfunction and both pulmonary hypertension and patent arterial duct.

Patent arterial duct is a common finding after birth asphyxia, as indicated in this study (62%). Its mechanism has been studied on the asphyxiated lambs.^18^ Patent arterial duct especially those with moderate to large size could be an additional factor to cause cardiac dysfunction and pulmonary hypertension as shown in our study.^16^,^17^,^19^ Tricuspid valve regurgitation was another prominent finding after birth asphyxia which was also reported in other studies.^4^ It was principally related to right ventricular or pulmonary hypertension and diastolic dysfunction (P<0.01). Papillary muscle ischemia in asphyxiated neonates also may cause tricuspid valve regurgitation such as those in adult patients with coronary artery disease.^4^,^10^

Our study showed high prevalence of pulmonary hypertension in asphyxiated neonates. Likewise, higher pulmonary pressure was associated with more hemodynamic instability. Pulmonary hypertension of neonates may play a role in rapid development of right and left ventricular dysfunction by their remodeling through reduced cardiolipin biosynthesis and remodeling enzymes.^20^,^21^

## CONCLUSIONS

Neonatal asphyxia causes a variety of cardiovascular disturbances including myocardial dysfunction and “transient pulmonary hypertension”. These hemodynamic abnormalities are real risks to the neonate; therefore, they deserve enough attention and early recognition for timely and appropriate management. Echocardiographic evaluation is warranted and the importance is emphasized
